# Association between health and safety perceptions of COVID‐19 vaccine and its uptake in Ghana

**DOI:** 10.1002/puh2.20

**Published:** 2022-10-13

**Authors:** Simon Appah Aram, John Elvis Hagan, George Kweku Afriyie Mansoh, Benjamin M. Saalidong, Patrick Osei Lartey, Bright Opoku Ahinkorah, Abdul‐Aziz Seidu, Edward Kwabena Ameyaw, Augustine Appiah, Divine Worlanyor Hotor, Justice Gyimah

**Affiliations:** ^1^ College of Safety and Emergency Management Engineering Taiyuan University of Technology Taiyuan People's Republic of China; ^2^ Department of Health Physical Education, and Recreation, University of Cape Coast, PMB Cape Coast Ghana; ^3^ Neurocognition and Action‐Biomechanics‐Research Group, Faculty of Psychology and Sport Sciences Bielefeld University Bielefeld Germany; ^4^ Department of Biochemistry and Biotechnology Kwame Nkrumah University of Science and Technology Kumasi Ghana; ^5^ Department of Geoscience and Engineering Taiyuan University of Technology Taiyuan People's Republic of China; ^6^ Key Laboratory of Interface Science and Engineering in Advanced Materials, Ministry of Education Taiyuan University of Technology Taiyuan People's Republic of China; ^7^ School of Public Health University of Technology Sydney Sydney Australia; ^8^ Centre for Gender and Advocacy Takoradi Technical University Takoradi Ghana; ^9^ College of Public Health, Medical and Veterinary Sciences James Cook University Townsville Australia; ^10^ CSIR Water Research Institute Accra Ghana; ^11^ College of Economics and Management Taiyuan University of Technology Taiyuan People's Republic of China

**Keywords:** Ghana, health, mandatory vaccination, safety, vaccine uptake, voluntary vaccination

## Abstract

**Background:**

Attitudes towards vaccines have affected COVID‐19 vaccination programs in many countries. This study sought to evaluate the effects of general perceptions on the safety and health concerns and the confidence in COVID‐19 vaccines on its uptake in Ghana.

**Methods:**

A cross‐sectional online survey was conducted between January and March 2021. The outcome variables for this study were “Taking mandatory COVID‐19 vaccine” and “Taking voluntary COVID‐19 vaccine”. The data were subjected to both descriptive (frequency, percentages, and chi‐square tests) and inferential (complementary log‐log logistic regression) analyses.

**Results:**

Out of 620 Ghanians who participated in the survey, about 80% of the participants believed that vaccines were good for one’s health and 73% had confidence on COVID‐19 vaccine safety; although 81% of the respondents were particularly concerned about the source of the vaccine. 79% and 71% of respondents indicated their willingness for mandatory and voluntary COVID‐19 vaccination, respectively. In all operationalized regression models, Ghanaians who believed that vaccines are healthy (OR = 1.998, Cl = 1.345–2.968; OR = 1.652, Cl = 1.050–2.601) and those who had confidence in a COVID‐19 vaccine safety (OR = 4.405, Cl = 3.136–6.188; OR = 8.340, Cl = 5.471–12.713) were more likely to take a mandatory or voluntary COVID‐19 vaccine compared to those who thought and believed otherwise. Individual preferences and/or intentions towards COVID‐19 vaccine uptake and uptake route (i.e., mandatory, voluntary) were influenced by multifaceted determinants: biosocial (age, marital status, education), socio‐cultural (religion, source of vaccine as a concern), and location (geographical zone) factors.

**Conclusion:**

To consolidate and possibly increase vaccine uptake in response to the COVID‐19 pandemic in Ghana, health education and promotion programs should aim at creating awareness on the benefits of vaccine uptake while addressing the health and safety concerns on the potential side effects through evidence‐based community messaging from credible sources. It is important to show specific commitment to transparency and reliable information to build public trust by decision‐makers.

## Graphical abstract

This study sought to evaluate the effects of general vaccine health perceptions and the confidence in COVID‐19 vaccine safety towards uptake in Ghana. The findings showed that about 80% of participants believed that vaccines were good for the health and 73% had confidence in a COVID‐19 vaccine safety, although 81% of the respondents were particularly concerned about the source of the vaccine. To consolidate and possibly increase vaccine uptake in response to the COVID‐19 pandemic in Ghana, health education and promotion programs should aim at creating awareness on the benefits of vaccine uptake while addressing the health and safety concerns on the potential side effects through evidence‐based community messaging from credible sources.

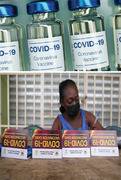

## BACKGROUND

The novel coronavirus, declared a global pandemic by the World Health Organization (WHO), came with many uncertainties regarding its origin, nature and course. [1] The pandemic has seen countries roll out pandemic response and preparedness, the major components being the administration of vaccines. [2] Governments globally imposed lockdowns, physical distancing, regular washing of hands and mandatory wearing of nose masks all as part of measures to halt the spread of the coronavirus. [3, 4] As part of a global effort to save lives, there was an unprecedented effort worldwide to find an effective vaccine against the pandemic. Given the scale of its severity, the development phase of a potential vaccine was expected to be proceeded by large‐scale vaccination programs in order to attain herd immunity against the virus. [5]

Finding an effective and safe vaccine for COVID‐19 would certainly be of great value to the fight against the pandemic. However, as the vaccines become available to the population, there are concerns on the uptake of these vaccines even as governments tend to prioritize health workers and other selected groups for the vaccination program. [5] In the event that there are enough vaccines in circulation, it does not automatically correspond to its acceptance by the public. The acceptance and usage of anti‐H1NI vaccine in 2009 was low. [6] The willingness to accept or reject vaccination is a major factor for the success of any vaccination program. It would be interesting to know the general reaction of the population towards voluntary or mandatory vaccine uptake. Varying responses to the willingness to accept COVID‐19 vaccine have been observed in parts of Europe. [5] Although many respondents reacted positively to vaccination, not all were in agreement. A model of determinants of vaccine hesitancy influences vaccine acceptance. Based on this premise, various explanations have been cited to influence a person's decision to accept or reject a COVID‐19 vaccine. [5, 7–9]

First is vaccine hesitancy, which is a behaviour influenced by issues of confidence, complacency and convenience. Vaccine hesitant individuals are a mixed group of people who hold varying degrees of indecision about vaccination or specific ones. [9] Among individuals who have shown resistance to vaccination programs could be influenced by a variety of serious considerations that are not about competing biological risks. [2] Most of these considerations are much of social, economic, religious, or moral beliefs. Additionally, trust in the effectiveness and safety of vaccines as well as the health delivery system, including the reliability and competence of the health system and policy makers who decide which vaccines are major issues. A divergent opinion has always been observed in communities that feel unrepresented by authorities in policy making and thus lack confidence in authorities that makes the decision regarding vaccination. These peculiar issues define vaccine confidence, which exists on a continuum and determines an individual's decision to accept a vaccine. [9] Vaccine complacency exists where perceived risks of vaccine preventable infections are low and vaccination is not seen as necessary preventive action. This tendency can be influenced by under‐appreciation of the value of vaccine or lack of knowledge. [9]

Vaccine convenience, which also affects decision to vaccinate, bothers on the quality of the service, real or perceived and the degree to which vaccination services are delivered at a time and location and in a way that is considered appealing, affordable, convenient, and comfortable. [9, 10, 11] Vaccine decision making by a populace is a complex process with many factors directly or indirectly affecting such decisions with some more important in certain contexts, experience, and circumstances. [10, 12–14] There have been proven records to show that there exists a gap between intention and actual behaviour to get vaccinated. In Germany, for example, a study showed that willingness to take influenza vaccine was about 45%, whereas the actual intake was about 9.4%. [15] Vaccine acceptance among general population can be associated with perceived risk of the COVID‐19 vaccine, side effects, religious beliefs, and level of education among other factors.

For a vaccination program to be effective, it depends on wide vaccine uptake even for high efficacy vaccines. Therefore, it is important to understand the various factors that affect a person's willingness to get vaccinated or otherwise to establish effective public health strategies. [16, 17] Previous studies on vaccine acceptance (see [18], [19]) were conducted in the pre‐pandemic stage when no COVID‐19 vaccines were available and mainly focused on frontline healthcare professionals. Those literature may not provide a good indication of vaccine uptake because of changing public perceptions of the pandemic and the available vaccines as the situation evolves. [20] Few studies have been conducted in Ghana to ascertain the extent to which the general populace will accept a COVID‐19 vaccine. It will be useful to understand the Ghanaian view on the current COVID‐19 vaccination for the management and response of the outbreak. This study investigates the effects of vaccine health and safety perceptions on COVID‐19 vaccine uptake in the Ghanaian context. Findings from the study will help in the implementation of health education and promotion programs that create awareness on the benefits of vaccine uptake while addressing the health and safety concerns on the potential side effects through evidence‐based community messaging from credible sources.

## MATERIALS AND METHODS

### Study design, data collection and sampling

A cross‐sectional survey was employed in this study between January and March 2021. The questionnaire was disseminated and administered through social media such as Twitter, Facebook, and WhatsApp using google forms. Convenience sampling was used to select volunteer participants for this study. A pilot study was conducted by the research team among 60 selected Ghanaians to pre‐test the questionnaire. The pilot study was to improve the content of the questionnaire by contextualizing it to better fit the purpose of the study. In all, 620 responses were received and used for this study based on a 95% confidence interval and at a 4% error rate.

### Study variables

#### Outcome variables

The outcome variables for this study were “Taking mandatory COVID‐19 vaccine” and “Taking voluntary COVID‐19 vaccine.” For each of these variables, respondents were asked if (1) they were willing to take a COVID‐19 vaccine shot if it is a requirement for a process or activity and (2) they were willing to voluntarily take a COVID‐19 vaccine. Response were given as “Yes” and “No” for each question.

#### Key predictors

The key predictors were “Belief that vaccines are healthy” and “Confidence in a COVID‐19 vaccine.” Participants were asked if they (1) believed that vaccines were generally healthy and (2) had confidence in a COVID‐19 vaccine safety. A response of “Yes” for any of the two questions meant that the participant believed that vaccines were generally healthy and also a potential COVID‐19 vaccine was safe. A “No” response implied otherwise.

#### Compositional and location variables

**FIGURE 1:**
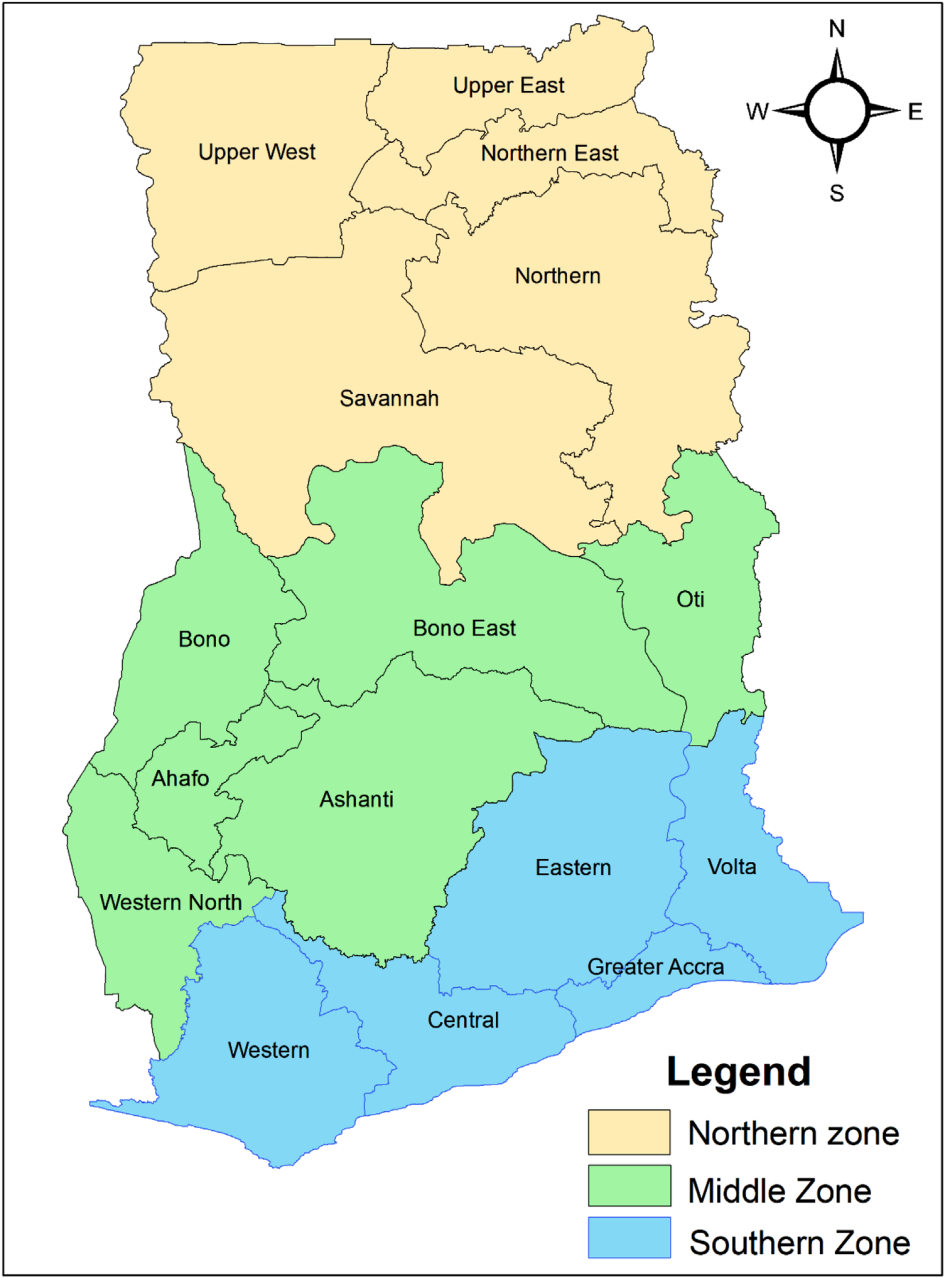
A map showing the geographical zones

The compositional factors denote the social and demographic attributes of the respondents. The compositional attributes in this case were the biosocial and sociocultural factors of the respondents. Biosocial factors are attributes present at birth and cannot be changed because of its intrinsic physical and biological components. This included gender (male or female) and age (18–24, 25–34, 35–50). The sociocultural attributes (values, customs, habits, beliefs etc. of the people) selected for this study were marital status (single or married), educational level (No formal education, Senior High, Tertiary), employment status (No or Yes), religion (Christianity, Islam, Others) and concern about vaccine source (No or Yes). Ghana was divided into three zones; Northern zone (Northern, Upper East, Upper West, Savannah and North East Regions), Middle zone (Ashanti, Ahafo, Oti, Western North, Bono East and Bono Regions), and the Southern zone (Western, Central, Greater Accra, Eastern and Volta Regions). These zones (see Figure 1) were created based on social, economic, and geographical similarities and for parsimony.

### Data analyses

The data analyses were done using Stata 15 MP (StataCorp, College Station, TX, USA) at a statistical significance of 0.05 and at a confidence interval of 95%. Descriptive analysis was used to examine the distribution of characteristics of respondents, vaccine health and safety perception and COVID‐19 vaccine uptake. Univariate, bivariate and complementary log‐log logistic regression statistical techniques were employed to assess associations between the dependent variables (“taking mandatory COVID‐19 vaccine” and “taking voluntary COVID‐19 vaccine”) and the explanatory variables. Three models were run; key predictors + biosocial (model 1), socio‐cultural (model 2), and location (model 3).

## RESULTS

### Respondents, health and safety perceptions and vaccine uptake

**FIGURE 2:**
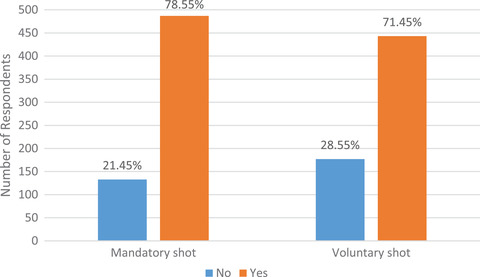
Percentage distribution of taking a mandatory and voluntary COVID‐19 vaccine by respondents

Table 1 shows the descriptive results. Male participants were 53% while female participants were 47%. Most of the respondents were aged 25–34 (58%). Participants who believed that vaccines were healthy were 80% while the remaining 20% believed that vaccines were not healthy. Also 73% had confidence in the safety of a COVID‐19 vaccine. However, 81% of the respondents were particularly concerned about the source of the vaccine. As shown in Figure 2, 79% of respondents indicated they will take a mandatory COVID‐19 vaccine while the remaining 21% indicated they will not take a mandatory COVID‐19 vaccine. Also, 71% of participants indicated they will take a voluntary COVID‐19 vaccine shot while the remaining 29% gave a dissented response. The reason why most of the participants were aged 18–34, had a tertiary education, were single and from the Southern zone is supported by the use of online survey in this study, where majority of those who responded to the surveys had access to social media platforms. Few older, married, those with no formal education and those in the Northern part of Ghana may not have access to social media platforms.

**TABLE 1:** Demographic characteristics of respondents

Variables	Frequency (*n*)	Percentage (%)
**Gender**		
Male	329	53
Female	291	47
**Age**		
18–24	179	29
25–34	358	58
35–50	43	7
Above 50	40	6
**Marital status**		
Single	508	82
Married	112	18
**Education**		
No formal	54	9
Senior High School (SHS)	81	13
Tertiary	485	78
**Religion**		
Christianity	499	80
Islam	105	17
Others	16	3
**Employment status**		
No	343	55
Yes	277	45
**Concern about vaccine source**		
No	67	11
Yes	553	89
**Country zone**		
Southern	489	79
Middle	67	11
Northern	64	10
**Belief that vaccine is healthy**		
No	122	20
Yes	498	80
**Confidence in COVID‐19 vaccine safety**		
No	167	27
Yes	453	73

### Predictors of taking a mandatory versus voluntary COVID‐19 shots

Table 2 presents Pearson's chi‐square test of independence. Pearson's chi‐square test was used to determine whether the observed differences in taking a mandatory shot and voluntary shot and, biosocial, as well as sociocultural and location factors were independent. For mandatory COVID‐19 vaccine shot, belief that vaccine is healthy (*χ* ^2^ = 150.3731, *p* < 0.001) and having confidence in COVID‐19 vaccine safety (*χ* ^2^ = 219.4854, *p* < 0.001) had statistically significant association with mandatory COVID‐19 vaccine shot. For the biosocial factors, gender and age had no statistically significant association with taking mandatory COVID‐19 vaccine shot. Of the sociocultural factors, religion (*χ* ^2^ = 8.1482, *p* ≤ 0.05) and vaccine source (*χ* ^2^ = 9.2048, *p* ≤ 0.005) had statistically significant association with taking mandatory COVID‐19 vaccine shot. Country zones (*χ* ^2^ = 9.2048, *p* = < 0.001) was statistically significant with taking mandatory COVID‐19 vaccine shot. For voluntary COVID‐19 vaccine shot, also in Table 2, belief that vaccine is healthy (*χ* ^2^ = 125.9256, *p* < 0.001) and having confidence in COVID‐19 vaccine safety (*χ* ^2^ = 278.9495, *p* < 0.001) had statistically significant association with voluntarily taking COVID‐19 vaccine shot. Among the sociocultural factors, only religion (*χ* ^2^ = 10.9674, *p* < 0.05) had statistically significant association with voluntarily taking a COVID‐19 vaccine shot. Furthermore, country zones (*χ* ^2^ = 9.9530, *p* < 0.005) also demonstrated statistically significant difference with taking voluntary COVID‐19 vaccine shot.

**TABLE 2:** Percentage distribution of taking a mandatory and voluntary COVID‐19 shot by predictor variables

	Mandatory shot		Voluntary shot	
Variables	No (%)	Yes (%)	No (%)	Yes (%)
**Vaccine is healthy** *χ* ^2^ (1) = 150.3731, *p* < 0.001			*χ* ^2^ (1) = 125.9256, *p* < 0.001	
No	76 (62.3)	46 (37.7)	85 (69.67)	37 (30.33)
Yes	57 (11.45)	441 (88.55)	92 (18.47)	406 (81.53)
**Vaccine is safe** *χ* ^2^ (1) = 219.4854, *p* < 0.001			*χ* ^2^ (1) = 278.9495, *p* < 0.001	
No	103 (61.68)	64 (38.32	131 (78.44)	36 (21.56)
Yes	30 (6.62)	423 (93.38)	46 (10.15)	407 (89.85)
**Gender** *χ* ^2^ (1) = 0.7523, *p* = 0.386			*χ* ^2^ (1) = 0.7698, *p* = 0.380	
Male	75 (22.8)	254 (77.20)	89 (27.05)	240 (72.95)
Female	58 (19.93)	233 (80.07)	88 (30.24)	203 (69.76)
**Age** *χ* ^2^ (3) = 3.5741, *p* = 0.311			*χ* ^2^ (3) = 5.7949, *p* = 0.122	
18–24years	42 (23.46)	137 (76.54)	53 (29.61)	126 (70.39)
25–34years	78 (21.29)	280 (78.21)	108 (30.17)	250 (69.83)
35–50years	9 (20.93)	34 (79.07)	11 (25.58)	32 (74.42)
Above 50 years	4 (10)	36 (90)	5 (12.5)	35 (87.50)
**Marital status** *χ* ^2^ (1) = 0.0681, *p* = 0.794			*χ* ^2^ (1) = 1.9067, *p* = 0.167	
Single	110 (21.65)	398 (78.35)	151 (29.72)	357 (70.28)
Married	23 (20.54)	89 (79.46)	26 (23.21)	86 (76.79)
**Education** *χ* ^2^ (2) = 3.1645, *p* = 0.206			*χ* ^2^ (2) = 5.8068, *p* = 0.055	
No formal	9 (16.67)	45 (83.33)	8 (14.81)	46 (85.19)
SHS/VOC	23 (28.40)	58 (71.6)	22 (27.16)	59 (72.84)
Tertiary	133 (21.45)	487 (78.55)	147 (30.31)	338 (69.69)
**Religion** *χ* ^2^ (2) = 8.1482, *p* ≤ 0.05			*χ* ^2^ (2) = 10.9674, *p* < 0.05	
Christianity	114 (22.85)	385 (77.15)	148 (29.66)	351 (70.34)
Islam	13 (12.38)	92 (87.62)	20 (19.05)	85 (80.95)
Other	6 (37.50)	10 (62.50)	9 (56.25)	7 (43.75)
**Employment**	χ^2^ (1) = 3.5536, *p* = 0.059		*χ* ^2^ (1) = 1.1215, *p* = 0.290	
No	64 (18.66)	279 (81.34)	92 (26.82)	251 (73.18)
Yes	69 (24.91)	208 (75.09)	85 (30.69)	192 (69.31)
**Vaccine source** *χ* ^2^ (1) = 9.2048, *p* ≤ 0.005 *χ* ^2^ (1) = 1.2303, *p* = 0.267				
No	24 (35.82)	43 (64.18)	23 (34.33)	44 (65.67)
Yes	109 (19.71)	444 (80.29)	154 (27.85)	399 (72.15)
**Zones** *χ* ^2^ (2) = 9.2048, *p* ≤ 0.001			χ^2^ (2) = 9.9530, *p* < 0.005	
Southern	124 (25.36)	365 (74.64)	152 (31.08)	337 (68.92)
Middle	6 (8.96)	61 (91.04)	17 (25.37)	50 (74.63)
Northern	3 (4.69)	61 (95.31)	8 (12.50)	56 (87.50)

### Bivariate logistic regression of taking mandatory COVID‐19 vaccine shot and predictor variables

For the key predictors in the bivariate analysis in Table 3, belief that vaccine is healthy and having confidence in a COVID‐19 vaccine safety were statistically significant in predicting taking mandatory COVID‐19 vaccine shot. Ghanaians who believed that vaccines are healthy and Ghanaians who had confidence in the safety of a COVID‐19 vaccine were 4.580 and 5.617 times, respectively, more likely to take a mandatory COVID‐19 vaccine shot. Also, Ghanaians who were above 50 years were 1.588 times more likely to take a mandatory COVID‐19 vaccine shot as compared to their 18–24 years counterparts. Likewise, Muslims were 1.415 times more likely to take a mandatory COVID‐19 vaccine shot as compared to Christians. Ghanaians from the middle and northern zones were 1.759 and 2.230 times more probable to take a mandatory COVID‐19 vaccine shot as compared to those from the southern zone, respectively.

**TABLE 3:** Bivariate complementary log‐log regression of taking mandatory and voluntary COVID‐19 vaccine shot

	Mandatory shot					Voluntary shot				
Variables	OR	Robust SE	*p*‐Value	Conf. interval		OR	Robust SE	*p*‐Value	Conf. interval	
**Vaccine is healthy (ref: No)**										
Yes	4.580	0.731	**<0.001**	3.349	6.263	4.673	0.816	**<0.001**	3.319	6.580
**Vaccine is safe (ref: No)**										
Yes	5.617	0.798	**<0.001**	4.252	7.421	9.420	1.677	**<0.001**	6.645	13.354
**Gender (ref: Male)**										
Female	1.091	0.109	0.385	0.896	1.327	0.915	0.093	0.381	0.749	1.117
**Age (ref: 18‐24years)**										
25‐34 years	1.051	0.120	0.662	0.841	1.314	0.985	0.114	0.894	0.784	1.236
35‐50years	1.079	0.228	0.720	0.713	1.632	1.120	0.239	0.595	0.738	1.701
Above 50 years	1.588	0.359	**0.041**	1.019	2.475	1.709	0.380	**0.016**	1.105	2.643
**Marital status (ref: Single)**										
Married	1.035	0.134	0.793	0.802	1.334	1.204	0.157	0.155	0.932	1.555
**Education (ref: No formal)**										
SHS/VOC	0.703	0.155	0.109	0.456	1.082	0.683	0.151	0.084	0.443	1.052
Tertiary	0.876	0.157	0.459	0.616	1.244	0.625	0.113	**0.009**	0.439	0.890
**Employed (ref: No)**										
Yes	0.828	0.083	0.061	0.679	1.009	0.898	0.092	0.291	0.735	1.097
**Religion (ref: Christianity)**										
Islam	1.415	0.193	**0.011**	1.083	1.848	1.364	0.183	**0.020**	1.049	1.774
Others	0.664	0.222	0.221	0.345	1.278	0.473	0.184	0.054	0.221	1.012
**Concern of vaccine source (ref: No)**										
Yes	1.582	0.266	**0.006**	1.138	2.199	1.196	0.200	0.285	0.862	1.659
**Zones (ref: Southern)**										
Middle	1.759	0.301	**0.001**	1.257	2.460	1.174	0.192	0.327	0.852	1.617
Northern	2.230	0.430	**<0.001**	1.528	3.255	1.780	0.301	**0.001**	1.277	2.480

Bold values are significant at <0.05.

### Bivariate logistic regression of taking voluntary COVID‐19 vaccine shot and predictor variables

From Table 3, belief that vaccine is healthy and having confidence in the safety of a COVID‐19 vaccine were statistically significant in predicting taking voluntary COVID‐19 vaccine shot. Ghanaians who believed that vaccines are healthy and had confidence in the safety of a COVID‐19 vaccine were 4.673 and 9.420 times more likely to take a voluntary COVID‐19 vaccine shot, respectively. Additionally, Ghanaians who were above 50 years were 1.709 times more likely to take a voluntary COVID‐19 vaccine shot as compared to their 18–24 years counterparts. Counterintuitively, Ghanaians with tertiary education were 38% less likely to take a voluntary COVID‐19 vaccine shot as compared to those with no formal education. Muslims were also 1.364 times more likely to take voluntary COVID‐19 vaccine shot as compared to Christians. Ghanaians from the northern zone were 1.780 times more likely to take a voluntary COVID‐19 vaccine shot as compared to those from the southern zone.

### Multivariate complementary log‐log regression model predicting taking mandatory COVID‐19 vaccine shot

Table 4 is a complementary log‐log multivariate logistic regression showing the three models; key predictors + biosocial model, sociocultural model, and the location model for predicting taking mandatory COVID‐19 vaccine shot in Ghana. In model 1 (key predictors+ biosocial), belief that vaccine is healthy, and having confidence in a COVID‐19 vaccine safety were statistically significant predictors of taking mandatory COVID‐19 vaccine shot. Ghanaians who believed that vaccines are healthy, and Ghanaians who had confidence in the safety of a COVID‐19 vaccine were 2.047 and 4.056 times more probable to take a mandatory COVID‐19 vaccine shot, respectively. Of the biosocial factors, Ghanaians who were between the ages 25 and 34 years were 1.345 times more likely to take a mandatory COVID‐19 vaccine shot as compared to those who were between 18 and 24 years.

**TABLE 4:** Multivariate complementary log‐log regression model predicting taking mandatory COVID‐19 vaccine shot

Variables	Model 1: Key predictors + Biosocial factors					Model 2: Key predictors + Biosocial factors + Socio cultural factors					Model 3: Key predictors + Biosocial factors + Socio cultural factors + Location Factor				
Legal requirement shot	OR	Robust SE	*p*‐Value	Conf. interval		OR	Robust SE	*p*‐Value	Conf. interval		OR	Robust SE	*p*‐Value	Conf. interval	
**Vaccine is healthy (ref: No)**															
Yes	2.047	0.383	**<0.001**	1.419	2.953	2.060	0.406	**<0.001**	1.400	3.031	1.998	0.403	**0.001**	1.345	2.968
**Vaccine is safe (ref: No)**															
Yes	4.056	0.678	**<0.001**	2.923	5.628	4.220	0.714	**<0.001**	3.028	5.881	4.405	0.764	**<0.001**	3.136	6.188
**Gender (ref: Male)**															
Female	1.241	0.159	0.092	0.966	1.594	1.281	0.171	0.064	0.986	1.664	1.301	0.179	0.055	0.994	1.703
**Age (ref: 18–24years)**															
25‐34 years	1.345	0.190	**0.035**	1.020	1.773	1.400	0.225	**0.036**	1.022	1.918	1.394	0.229	**0.043**	1.011	1.924
35‐50years	1.486	0.373	0.114	0.909	2.430	1.415	0.382	0.199	0.833	2.401	1.289	0.352	0.352	0.755	2.201
Above 50 years	1.625	0.444	0.075	0.952	2.775	1.240	0.479	0.577	0.582	2.642	1.274	0.478	0.519	0.610	2.659
**Marital status (ref: Single)**															
Married						1.115	0.193	0.530	0.794	1.564	1.130	0.207	0.507	0.789	1.618
**Education (ref: No formal)**															
SHS/VOC						0.698	0.208	0.229	0.389	1.253	0.663	0.187	0.146	0.381	1.154
Tertiary						0.717	0.207	0.248	0.407	1.261	0.624	0.170	0.084	0.365	1.066
**Employed (ref: No)**															
Yes						0.865	0.114	0.273	0.668	1.120	0.894	0.122	0.409	0.684	1.167
**Religion (ref: Christianity)**															
Islam						1.637	0.239	**0.001**	1.230	2.178	1.384	0.247	0.069	0.974	1.964
Others						1.371	0.494	0.382	0.676	2.778	1.190	0.462	0.654	0.556	2.549
**Concern about vaccine source (ref: No)**															
Yes						1.474	0.283	**0.043**	1.012	2.146	1.592	0.326	**0.023**	1.066	2.377
**Zones (ref: Southern)**															
Middle											2.334	0.558	**<0.001**	1.461	3.728
Northern											1.832	0.587	0.059	0.978	3.431

Bold values are significant at <0.05.

In model 2, where sociocultural factors were controlled for, belief that vaccine is healthy and having confidence in a COVID vaccine safety were still statistically significant predictors of taking a mandatory COVID‐19 vaccine shot. Age was still the only significant predictor among the biosocial factors. Of the sociocultural factors, Muslims were 1.637 times more likely to take a mandatory COVID‐19 vaccine shot as compared to their Christian counterparts. Likewise, Ghanaians who were concerned about the source of a COVID‐19 vaccine were 1.474 times more likely to take a mandatory COVID‐19 vaccine shot as compared to those who did not care where the vaccine came from.

When location was controlled for in model 3, belief that vaccine is healthy and having confidence in the safety of a COVID vaccine were robust and persisted in predicting taking a mandatory COVID‐19 vaccine shot. Ghanaians who believed that vaccines are healthy and had confidence in a COVID‐19 vaccine safety were 1.998 and 4.405 times, respectively, more likely to take a mandatory COVID‐19 vaccine shot as compared to Ghanaians who did not believe vaccines are healthy and also did not trust the safety of a COVID‐19 vaccine. Of the biosocial and sociocultural factors, only age and vaccine source were significant predictors. Ghanaians who were 25–34 years were 1.394 times more likely to take a mandatory COVID‐19 vaccine shot as compared to those who were 18–24 years. Similarly, Ghanaians who were concerned about the source of a potential COVID‐19 vaccine were 1.592 times more likely to take a mandatory COVID‐19 vaccine shot as compared to those who were not bothered about the source of a COVID‐19 vaccine. For the location factor, Ghanaians from the middle zone were 2.334 times more likely to take a mandatory COVID‐19 vaccine shot as compared to those in the southern zone.

### Multivariate complementary log‐log regression model predicting taking voluntary COVID‐19 vaccine shot

Table 5 is also a complementary log‐log multivariate logistic regression showing the three models; key predictors + biosocial model, sociocultural model, and the location model for predicting taking voluntary COVID‐19 vaccine shot. In model 1 (key predictors+ biosocial), only confidence in COVID‐19 vaccine safety was a statistically significant predictor of taking voluntary COVID‐19 vaccine shot among the key predictors. In this instance, Ghanaians who had confidence in a COVID‐19 vaccine safety were 7.856 times more likely to take a voluntary COVID‐19 vaccine shot. None of the biosocial factors was statistically significant in predicting taking voluntary COVID‐19 vaccine shot.

**TABLE 5:** Multivariate complementary log‐log regression model predicting taking voluntary COVID‐19 vaccine shot

Variables	Model 1: Key predictors + Biosocial factors					Model 2: Key predictors + Biosocial factors + Socio cultural factors					Model 3: Key predictors + Biosocial factors + Socio cultural factors + Location Factor				
Voluntary shot	OR	Robust SE	*p*‐Value	Conf. interval		OR	Robust SE	*p*‐Value	Conf. interval		OR	Robust SE	*p*‐Value	Conf. interval	
**Vaccine is healthy (ref: No)**															
Yes	1.508	0.324	0.055	0.99	2.297	1.680	0.387	**0.024**	1.07	2.639	1.652	0.382	**0.030**	1.050	2.601
**Vaccine is safe (ref: No)**															
Yes	7.856	1.589	**<0.001**	5.285	11.676	8.373	1.798	**<0.001**	5.496	12.756	8.340	1.794	**<0.001**	5.471	12.713
**Gender (ref: Male)**															
Female	0.825	0.100	0.114	0.651	1.047	0.781	0.099	0.051	0.609	1.001	0.787	0.099	0.057	0.615	1.008
**Age (ref: 18–24years)**															
25‐34 years	1.125	0.151	0.378	0.865	1.464	1.282	0.196	0.105	0.949	1.731	1.301	0.201	0.088	0.961	1.761
35‐50years	1.319	0.331	0.271	0.806	2.157	1.193	0.347	0.544	0.674	2.111	1.209	0.353	0.516	0.682	2.144
Above 50 years	1.62	0.427	0.067	0.966	2.715	0.697	0.288	0.383	0.310	1.567	0.703	0.293	0.398	0.311	1.591
**Marital status (ref: Single)**															
Married						1.450	0.259	**0.037**	1.022	2.058	1.438	0.258	**0.043**	1.012	2.045
**Education (ref: No formal)**															
SHS/VOC						0.481	0.184	0.056	0.227	1.019	0.457	0.175	**0.041**	0.216	0.967
Tertiary						0.294	0.105	**0.001**	0.146	0.592	0.265	0.095	**<0.001**	0.131	0.537
**Employed (ref: No)**															
Yes						0.864	0.112	0.261	0.67	1.115	0.884	0.115	0.341	0.685	1.140
**Religion (ref: Christianity)**															
Islam						0.99	0.171	0.954	0.706	1.389	0.845	0.158	0.366	0.586	1.218
Others						0.933	0.248	0.793	0.554	1.569	0.936	0.246	0.801	0.558	1.568
**Concern about vaccine source (ref: No)**															
Yes						1.088	0.211	0.661	0.745	1.590	1.093	0.210	0.642	0.751	1.592
**Zones (ref: Southern)**															
Middle											1.054	0.194	0.776	0.734	1.513
Northern											1.357	0.325	0.202	0.849	2.169

Bold values are significant at <0.05.

In model 2, where sociocultural factors were controlled for, confidence in vaccine safety was still statistically significant. A new relationship however emerged, indicating mediation by the sociocultural factors. Ghanaians who believed that vaccine is healthy were 1.680 times more likely to take a voluntary COVID‐19 vaccine shot as compared to those believe vaccines are not healthy. None of the biosocial factors were still statistically significant predictors. Among the sociocultural factors, those who were married were 1.450 times more probable to take a voluntary COVID‐19 vaccine shot as compared to their single counterparts. Counterintuitively, Ghanaians with tertiary education were 71% less likely to take a voluntary COVID‐19 vaccine shot as compared to those with no formal education.

When location was controlled for in model 3, belief that vaccine is healthy and having confidence in the safety of a COVID vaccine were robust and persisted in predicting taking voluntary COVID‐19 vaccine shot. Ghanaians who believed that vaccines are healthy and had confidence in a COVID‐19 vaccine safety were 1.652 and 8.340 times, respectively, more likely to take a voluntary COVID‐19 vaccine shot as compared to Ghanaians who did not believe vaccines are healthy and also did not trust the safety of a COVID‐19 vaccine. Of the biosocial and sociocultural factors only marital status and education were significant predictors. Ghanaians who were married were 1.438 times more likely to take a voluntary COVID‐19 vaccine shot as compared to their single counterparts. Counterintuitively, Ghanaians with tertiary education and senior high education were 54% and 74% less likely to take a voluntary COVID‐19 vaccine shot as compared to those with no formal education, respectively. The location factor was not a significant predictor.

## DISCUSSION

A vaccine against COVID‐19 has been suggested as an effective strategy to end the pandemic. [21, 22] Despite this epidemiological knowledge, only few studies, especially in sub‐Saharan Africa have explicitly investigated perceptions on vaccines and which vaccination approach would yield positive uptake. This contribution investigated the effects of vaccine health and safety perceptions on COVID‐19 vaccine uptake in Ghana. Understanding people's perceptions and preferences for COVID‐19 vaccination could help guide public health policy measures on the vaccination rollout in the country.

Preliminary findings found evidence that 80% and 73% of participants believed that vaccines were healthy and safe, although 81% of the respondents were particularly concerned about the source of the vaccine. Other evidence revealed that 79% and 71% of respondents indicated their willingness for mandatory and voluntary COVID‐19 vaccine uptake or shot, respectively. These observed rates are comparable with other studies (i.e., ranging from 60% to 90%) (e.g., [23]–[26]). Current findings indicate that majority of the studied population are supportive of the COVID‐19 vaccine in the country regardless of the uptake approach (either mandatory or voluntary). This pattern observed is not surprising because the study was conducted around the period (i.e., January–March 2021) when the number of COVID‐19 cases in Ghana were on a steady rise; thus, vaccines were seen as a key strategy to halt the escalation of the COVID19 pandemic. Overall, the observed figures suggest a positive attitude towards COVID‐19 vaccination that could hinge on the highly reported pandemic impact across many societies and perceive benefits (e.g., reduce risk) the vaccines may bring.

Although majority of the studied sample will either take mandatory or voluntary COVID‐19 vaccine shot, small proportions (i.e., 21% and 29%) were unwilling to take a vaccine shot, perhaps due to safety concerns or vaccine uncertainty. Additionally, two‐thirds of the sample also had concern or uncertainty about the source of vaccines. This vaccine hesitancy and concern should be of public health interest because of the potential delays and/or outright refusal of vaccination across the population until the safety of vaccines is confirmed. Previous studies have often cited public concerns as a foremost barrier to vaccination decision making, especially for newly rolled out vaccines which have not been ecologically tested. [27–32]

Across all the regression models, confidence or belief and safety of vaccines predicted mandatory and/or voluntary COVID‐19 vaccine uptake of the studied Ghanaian population. This finding supports previous studies on public belief or trust on the health and safety or efficacy in the COVID‐19 vaccine as relevant factors that could increase COVID‐19 vaccine uptake (e.g., [10], [33, 34, 35]). Current evidence suggests the significance of enhancing public trust and belief in COVID‐19 vaccines and improving healthcare services to facilitate considerable vaccine uptake. This goal can be achieved through the use of trusted well‐tailored messages on COVID‐19 and confidence‐building advocacy on identified vaccines through transparency and expectation management [36]. For example, Tam et al. [37] reiterated that worries about vaccines’ long‐term side effects, safety issues and public distrust can lead to vaccine hesitancy. Hence, considerable community level engagements or interactions on vaccine related concerns for appropriate feedback should be done to counteract misinformation and/or disinformation as well as other biases against impending vaccine rollout [34, 38–40]. According to Schwartz [40] when public trust or confidence associated with COVID‐19 vaccination is weak, uptake programs are likely to suffer. Therefore, public messages on the vaccines’ safety and continuous monitoring as well as tackling of false information are crucial [38, 41, 42].

Increasing age, religion, and geographical zone increased the odds of COVID‐19 vaccine mandatory uptake. This finding mirrors similar trends that older people show more support for mandatory uptake compared to younger cohorts based on an established premise that case‐fatality rates increase with age [10, 43]. Alternatively, high perceived vulnerability and/or susceptibility to disease infection often associated with increasing age could also account for the present finding. [44]

Consistent with previous studies (e.g., [45, 46]), self‐reported religious affiliation was identified as significant factor in the determination of mandatory COVID‐19 vaccination uptake in the current study. Specifically, Muslims were 1.6 times more likely to take a mandatory COVID‐19 vaccine compared to Christians. The observed variation is not surprising because Christian religious concerns about immunization or vaccination date back to antiquity where some individuals prohibited Edward Jenner's 1796 mode of smallpox vaccination as contrary to God's will. [47] Some Christian denominations (e.g., Jehovah Witness) have a strong tradition of declining some health services like blood transfusion, including immunization on the concerns about their adverse effects similar to the happenings after smallpox vaccination during the 18th century. The basis for this objection by members of these denominations includes declining immunization instead of making members less dependent on God. [48–51] The noted differences in the COVID‐19 vaccine uptake between Muslim and Christian groups in the current study might not hinge on their religious beliefs, instead the variations may be reflections on safety and other health concerns. [52]

Study participants from the middle zone (e.g., Ashanti, Brong‐Ahafo, Western North Regions) of Ghana were 2.3 times more likely to take mandatory COVID‐19 vaccine than their counterparts from the southern zone (e.g., Greater Accra, Eastern and Volta Regions). Geo‐spatial metrics (e.g., population density) could possibly explain the current observation [53, 54]. Previous research has shown that vaccination rates may suffer among varied population groups, especially in areas of deprivation. [55–57] For example, individuals from less densely populated areas in the middle zone of the country might spend less time or waiting period for a vaccine uptake. Users are likely to perceive that uptake process as less stressful than respondents from highly densely populated areas such as Greater Accra, where access to healthcare services is often compounded by unfriendly and stressful population dynamics (e.g., long queues, huge traffic congestion, and long waiting hours). Additionally, respondents from geographical areas with readily available healthcare services and easily accessible facilities with considerable health logistical support (e.g., vaccination sites) are more likely to accept mandatory vaccine uptake compared to participants from other geographical boundaries with less endowed health infrastructure and logistics. Therefore, health inequities or disparities may restrict or negatively impact mandatory COVID‐19 vaccine uptake [58, 59]. It is important that major stakeholders address these population dynamics and resources that might be instrumental in facilitating comprehensive vaccine uptake in the country.

Other findings show that Ghanaians who are married were 1.438 times more likely to take a voluntary COVID‐19 vaccine shot compared to their single counterparts. Being married comes with more household interactions and connectedness. Hence, we speculate that those married might have additional responsibility of protecting the entire family due to high‐risk perception and would show more willingness or positive intention towards taking COVID‐19 vaccine, though voluntarily. Previous research has cited social interaction and connectedness as important risk factors of COVID‐19 infection [60, 61]. Raising continuous awareness about the risk of COVID‐19 infection, especially among the unmarried population is essential towards reducing case fatality [35].

Counterintuitively, participants with tertiary education were 71% less likely to take a voluntary COVID‐19 vaccine shot compared to their counterparts with no formal education. This finding demonstrates the complexity of infectious disease dynamics: an observation that overrides conventional standards in epidemiological assessment. Individuals’ risk perception may determine one's intention to get vaccinated against COVID‐19, with those perceiving a higher risk towards COVID‐19 more likely to show the intention to voluntarily vaccinate against the virus. It is likely that sampled tertiary educated individuals might have less risk perception against the new virus; hence, their intention to voluntarily vaccinate is low. For individuals with no educational background, the new virus may create enormous psychological distress triggered by worrying concerns and fears that could heighten their voluntary intention to take a vaccine shot [62, 63, 64, 65]. The current finding also mirrors the risk as feelings model that confirms the role risk perception plays on judgement and decision making in health care for diseases of severe magnitude and uncertain outcome, demonstrating how one's perceived risk might influence the decision to vaccinate (see [66, 67, 68]). Therefore, the general public ought to realize the severity of COVID‐19, hence underestimating their risks of contracting the virus may prevent them from being vaccinated. [69] Current finding implies that considerable efforts ought to be targeted at those in the population with the highest severity [70]. Regular educational campaigns to promote COVID‐19 vaccines should target personal risks to the disease through persuasive communication in the general population.

### Strengths and limitations

This is the first study to provide estimates on individual preferences relative to their predictors associated with COVID‐19 vaccine uptake across a cross‐section of the population in Ghana. Current findings advance theoretical knowledge by providing a better understanding in the context of COVID‐19 health policies. Empirical evidence provided can aid stakeholders on which COVID‐19 vaccination policy to implement. Despite these strengths, the present study has some limitations.

Preferences and/or intentions are hypothetical scenarios that vary from real life behaviours; consequently, it is likely that individual reactions to real life COVID‐19 events might be stronger such that our current findings could be seen as conservative approximations [71, 72]. Additionally, it is also possible that the responses during the data collection were relative to time, with the possibility of change over patterns in intentions and subsequent action tendencies once the vaccines were made available [73]. For example, COVID‐19 vaccine rollout had not begun at the time of the data collection (i.e., actual vaccine uptake was not measured); hence, respondents’ real sense of judgement on vaccine uptake might not be conditioned by the resolutions taken later on the vaccines. Finally, due to the self‐reported nature of the study design, we cannot discount reporting biases. Since data were collected through an online survey via social media platforms, there is a possibility of biasness against those who had no access to social media, internet, and those who could neither read nor write. These people are mostly married, uneducated, older and live in the country‐side and parts of Northern sector of the country.

### Practical implications

Current evidence suggests that public belief or trust, uncertainties, health, and safety issues as well as socio‐cultural considerations surrounding COVID‐19 vaccines should guide future rollout programs. Despite present results indicating respondents’ positive attitude towards vaccine rollout spectrum (i.e., more restrictive: compulsory mandates and less restrictive options: opt‐in voluntarily), the current health emergency and the fluctuations in the epidemiological data (e.g., fatality and mortality metrics) leaves no room for low vaccine uptake. Based on observed findings, policies on vaccines in the country could provide a balance between the two different strategies for the administration of COVID‐19 vaccines. Therefore, designed interventions and support mechanisms are required for vaccine uptake in Ghana. Vaccination campaigns in the country should be based on scientific evidence on the vaccine efficacy, safety, and side effects made available to the public. Regular education, appropriate information and communication should target health and safety concerns associated with COVID‐19 vaccines. It is important that public trust and confidence are built through transparent and truthful communications on the vaccines. Developing vaccination initiatives through health technological interventions (e.g., providing regular telephone reminders, motivational text message reminders) and smart phone applications may consolidate and further boost positive vaccination behaviour and improve vaccination rates in the country.

## CONCLUSION

This present study provides evidence‐based variations in individual preferences and/intentions toward COVID‐19 vaccine uptake based on multifaceted determinants: biosocial (e.g., age, marital status, education), socio‐cultural (e.g., religion), location (e.g., geographical zone), and other important predictors (e.g., confidence in vaccine, vaccine health and safety, source of vaccine as a concern). Overall, attitude towards COVID‐19 vaccine uptake (i.e., mandatory, voluntary) was positive, though the odds of being vaccinated are influenced by the factors cited above. To consolidate and possibly increase vaccine uptake in response to the COVID‐19 pandemic in Ghana, health education and promotion programmes should aim at creating awareness on the benefits of vaccine uptake while addressing the health and safety concerns on the potential side effects through evidence‐based community messaging from credible sources. It is important to show specific commitment to transparency and reliable information to build public trust by decision‐makers. Future studies could integrate health behaviour theories in the prediction of COVID‐19 vaccine uptake and also observe how public intentions to vaccinate change over time over in Ghana.

## AUTHOR CONTRIBUTIONS

Simon Appah Aram, Patrick Osei Lartey, Augustine Appiah, Justice Gyimah and Divine Worlanyor Hotor designed the questionnaire, conducted the study, conducted statistical analyses. Simon Appah Aram, George Kweku Afriyie Mansoh, Benjamin M. Saalidong, Abdul‐Aziz Seidu and John Elvis Hagan wrote the first draft. Simon Appah Aram, John Elvis Hagan, Bright Opoku Ahinkorah, Abdul‐Aziz Seidu and Edward Kwabena Ameyaw contributed to the interpretation of data, revised the article and approved the final version. All authors have read and agreed to the published version of the manuscript.

## CONFLICT OF INTEREST

The authors declare that they have no competing interests.

## ETHICAL STATEMENT

Ethical review and approval was sought from the Taiyuan University of Technology Ethical Review Board (62/12/20). The online form required participants to read the background information of the study and then indicate whether they were willing to participate before they were able to proceed to respond to the questions. Also, participants received instant feedback on their responses. Participation was restricted to resident Ghanaian adults aged 18 years and above. Participants were not coerced or financially induced to take part in the study.

## Data Availability

The datasets of the current study are available from the corresponding author on reasonable request.
